# Body Composition Changes in College Athletes during the COVID-19 Lockdown

**DOI:** 10.23937/2469-5718/1510220

**Published:** 2022-04-24

**Authors:** Aston Dommel, Jose R Fernandez, R Drew Sayer

**Affiliations:** Department of Nutrition Sciences, University of Alabama at Birmingham, USA

## Abstract

**Background:**

In the spring of 2020, COVID-19 shocked the college sports world with athletes having seasons abruptly canceled and sent home under mandatory lockdown orders. Athletes and athletic performance staff had no idea when they would be back on campus or have access to on-campus athletics facilities. This situation caused substantial concern regarding potential adverse changes to athletic performance and body composition in the athletes. The purpose of this study is to assess how weight, muscle mass, and fat mass changed in collegiate athletes while they were prohibited from using on-campus athletic facilities due to the COVID-19 pandemic.

**Methods:**

Body weight, fat mass, and muscle mass were measured using bioelectrical impedance analysis as part of routine care for 77 collegiate athletes (n = 43 male, n = 34 female) pre-lockdown (Jan 2020) and shortly after their return to on-campus training (Aug/Sept 2020). 4 questions were asked to assess eating behavior and physical activity. Pre- and post-lockdown body composition data and survey data were analyzed using ANOVA and ANCOVA (SAS 9.4). To account for differences in body size, height was used to calculate Body Mass Index (BMI), Fat Mass Index (FMI), and muscle mass index (MMI) for assessing changes in weight, fat mass, and muscle mass, respectively.

**Results:**

No significant differences by sex in BMI or MMI were detected between pre and post lockdown. FMI changed according to sex, males lost FMI and females gained FMI.

**Conclusion:**

These data demonstrate potential sex differences in fat mass changes among college athletes during a mandatory absence from on-campus athletic facilities and in-person support from coaching and performance staff. Future research should determine whether future breaks - either anticipated or unanticipated-influence body composition and what the drivers of changes in body composition may be. Such research may help to develop sex-specific strategies for maintaining optimal body composition and athletic performance during extended breaks from structured athletic training.

## Introduction

College athletes train extensively for their sport over the course of the year. During in-season periods, these athletes train up to 20 hours a week with additional hours spent on recovery, for a total commitment of upwards of 30 hours a week. In the off-season, athletes still engage in at least 8 hours of training per week and continue to spend time on recovery. Mandatory breaks from intensive training are integrated into collegiate athletes’ training cycles to provide time to focus on academics and health. However, it is possible that athletes may experience reversals of body composition and performance gains during these breaks from training. The impact of mandatory breaks from training on body composition in collegiate athletes, especially unanticipated breaks such as during the COVID-19 lockdown, warrants further investigation.

COVID-19 had numerous adverse impacts on athletes’ mental and physical health including a struggle to return to play, difficulty maintaining adequate Physical Activity (PA), sleep pattern changes, lack of motivation, and overall less healthy behaviors [1,2]. These behavior changes could have resulted in detraining and reductions in performance [3]. Due to the novelty of the situation, researchers and athletics staff know very little about the consequences of the COVID-19 lockdown on athlete’s body composition. Previous studies that have investigated the impact of COVID-19 lockdowns on athlete body composition show conflicting results. Two studies, one in football players [4] with 4 months between pre- and post-COVID-19 measurements and a 2-month study in fencing athletes [5], both showed a gain of fat mass and loss of muscle mass. Alternatively, a study in soccer athletes showed no change in body composition [6] during 1 month in lockdown. These conflicting results could be partially driven by differences in the amount of time between pre- and post-COVID-19 body composition measurements. In particular, it is possible that 1 month of lockdown is not a sufficient amount of time to observe and detect body composition changes.

Body composition is important for performance and success in sports [7–10]. The absolute amount of fat and fat-free mass can impact performance by influencing both strength and speed [11]. Reductions in fat mass and increases in fat-free mass have been shown to increase power output and force generation leading to improved performances [12]. These changes in body composition are influenced by a variety of factors, mainly one’s PA regimen and diet [13,14]. Given that PA and diet are likely to change during extended breaks, particularly when unanticipated as in the COVID-19 lockdown, it is possible that body composition was adversely impacted during this time.

To date, little is known about body composition changes in athletes during the COVID-19 lockdown, and the data currently available in the literature are conflicting. It is important to understand how breaks from structured activity impact body composition in order to maximize player readiness and to understand the influence of diet and PA on these changes. The purpose of this study was to investigate changes in body composition during the COVID-19 lockdown and determine if body composition changes were different between male and female athletes. We also obtained question data from athletes on eating and physical activity behaviors to assess how these may have influenced body composition changes. We hypothesized that body composition would be worse (i.e., increased fat mass and reduced fat-free mass) upon the return from the COVID-19 lockdown and that there would be no sex differences body composition changes. We further hypothesized that unhealthy changes in diet (greater overall intake and reduced diet quality) and PA (reduced overall PA and reduced quality of PA) would at least partially explain changes in body composition.

## Methods

### Participants

Data for this study were obtained from student-athletes enrolled at a Southeastern division 1 university (University of Alabama at Birmingham, UAB). Body composition assessments are routinely conducted on many student-athletes, which were used for this analysis. Athletes eligible for inclusion in this analysis were those with valid body composition measures from Jan/Feb 2020 (pre-COVID-19 lockdown) and Aug/Sept 2020 (post-COVID-19 lockdown) and participated in the following sports: Baseball, softball, women’s basketball, men’s soccer, and beach volleyball. Student-athletes participating in football, men’s and women’s golf, men’s and women’s tennis, track and field, cross country, court volleyball, rifle, and men’s basketball were not eligible for inclusion in this analysis due to not routinely having body composition measured, different dates of measurement, or different methods for measuring body composition compared to the sports included.

### Protocol

Body composition data were collected on athletes prior to lockdown orders being implemented (January/February 2020) and as soon as athletes returned to the university setting (August/September 2020). Athletes also completed questions on changes in PA and diet during the lockdown period, which was investigated as potential mediators of changes in body composition. These questions were created by an author (AJD) who was a registered dietitian for UAB Athletics at the time of data collection. Body composition and question data were initially collected as part of routine care and were retrospectively approved for research use by the Institutional Review Board at the University of Alabama at Birmingham. Student athletes with complete body composition measures from pre- and post-lockdown provided informed consent to allow their data to be used for research purposes.

### Body composition assessment

Body composition and body weight were measured using Tanita MC-780U (Tanita Corp of America, Inc. Arlington Heights, Illinois, USA) a multi-current 8-mode bioelectrical impedance analysis machine. Outcomes recorded at baseline and follow-up included body weight, percent body fat, fat mass, muscles mass, fat free mass, and total body water percentage. Athletes were assessed in the morning upon waking in a fasted state with lightweight clothing and with socks and shoes removed.

### Self-reported changes in diet and PA behaviors questions

Changes in the perceived quality and quantity of diet and of PA during the lockdown compared to their usual behaviors were assessed using a 5-point Likert scale. For quality of diet and PA, the Likert scale was anchored with descriptions of 1: A lot worse and 5: A lot better. For quantity of diet and PA, the Likert scale was anchored with descriptions of 1: A lot more and 5: A lot less. Diet and PA quality responses of 1 (A lot worse) and 2 (Slightly worse) and 4 (Slightly better) and 5 (A lot better) were combined into single categories of “worse” and “better,” respectively, which resulted in 3 categories of “worse”, “no change”, or “better” diet and PA quality. Similarly, diet and PA quantity responses of 1 (A lot more) and 2 (Slightly more) and 4 (Slightly less) and 5 (A lot less) were combined into relevant categories resulting in 3 categories of diet and PA quantity (more, no change, and less). The questions available for reference in Appendix 1.

### Statistical analysis

Descriptive statistics were conducted to obtain mean and standard deviation for quantitative variables and frequency statistics for the diet and PA survey responses. Height was used to calculate BMI (kg body weight/m^2^), muscle mass index (MMI, kg muscle mass/m^2^), and fat mass index (FMI, kg fat mass/m^2^) to account for differences in overall body size among the athletes. Exploratory analyses were performed to identify potential confounders for the statistical models using correlation analysis with body composition variables and survey data. Primary models were initially used to test for differences in body composition changes by sex without including diet and PA behavior survey responses. This decision was made because these survey-based questions were initially created by AJD for non-research purposes and their inclusion in the analysis should be considered as exploratory. Initial, primary models included the following: Analysis of Variance (ANOVA) was used to test for differences in changes in BMI between male and female athletes. Sex differences between MMI and FMI change were assessed using analysis of covariance (ANCOVA) with FMI and MMI as covariates, respectively. Subsequently, more comprehensive ANCOVA models were used to test for sex differences in body composition changes with the inclusion of survey responses for diet and PA quality and quantity. In all of the models described above the dependent variable was body composition change and the independent variable was sex. Significance was set at p < 0.05 for all outcomes and SAS version 9.4 (Cary, NC) was used for all statistical analyses.

## Results

**Figure 1:**
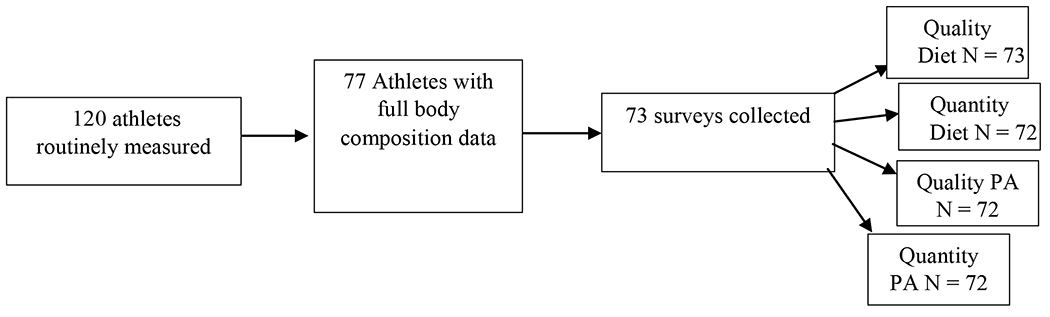
Flow chart for COVID-19 Lockdown recruitment.

As shown in Figure 1, N = 77 of the total 120 student athletes eligible for the study had complete body composition data from before and after the COVID-19 lockdown. Self-reported diet and PA data were provided by 73 of 77 athletes. Body composition characteristics of the n = 77 athletes are shown in Table 1.

Table 2 shows results from the initial minimally-adjusted models for sex differences and body composition change. The overall models for ΔFMI (F=8.79, p<.01) and ΔMMI (F=3.58 , p=0.03) were significant, whereas the overall model for ΔBMI was not significant (F=3.43, p=0.07). Over the lockdown, ΔFMI was different between men and women (F = 11.21, p < 0.01). ΔFMI was reduced in males (−0.22 ± 0.50), which is equivalent to approximately at 1% reduction in total fat mass. Alternatively, ΔFMI increased among females (0.28 ± 0.91) or approximately a 1% increase in fat mass. There was a trend for a sex difference in ΔBMI (F=3.43, p = 0.07) that appears to be driven by ΔFMI as there was no significant difference in ΔMMI by sex.

As shown in Table 3 the majority of athletes reported that the quality of their diet either did not change or improved during the COVID-19 lockdown, with only 20 athletes (27%) reporting worse diet quality. Half of the athletes reported eating more during the COVID-19 lockdown, while the other 50% reported either eating less or no change. Approximately half of athletes reported a decrease in both the quality and quantity of PA during the COVID-19 lockdown.

Table 4, Table 5 and Table 6 are results of more comprehensive models testing for sex differences in body composition changes with the inclusion of the survey responses of diet and PA quality and quantity. The overall models for ΔBMI (F=0.75, p = 0.66) and ΔMMI (F=1.61, p = 0.13 ) were not statistically significant and therefore results from these analyses are inconclusive. The overall model for ΔFMI was statistically significant (F=2.16, p = 0.03 ), but including diet and PA survey responses did not alter the observed sex difference in ΔFMI and no main effects were detected for diet or PA.

## Discussion

Results of this study partially support our hypothesis that body composition would be worse (i.e., increased fat mass and reduced fat-free mass) upon return from the COVID-19 Lockdown, but do not support our hypothesis that there would be no sex differences in body composition change. Changes in body composition differed by sex. During the COVID-19 Lockdown women gained fat mass, a negative change, while men lost fat mass, a positive change. Changes in fat mass by sex during the COVID-19 lockdown were not related to self-reported changes in diet and activity levels. No other significant relationships or changes by sex were seen in BMI or MMI.

Results of our study are somewhat different to what has been reported in the general population in response to the COVID-19 lockdown, which have found weight gain that was partially due to a decrease in activity [15,16]. Prior studies in athletes and non-athlete college students reported a gain in body fat and loss of muscle mass during COVID-19, despite a shorter window of assessment compared to our study (2 months vs. 6-7 months) [5,17,18]. The study of elite fencing athletes by Yasuda, et al. [5] showed no change in body composition for men but a gain in fat mass for women. In male soccer players a similar to the female fencing athletes fat mass increased leading to decreases in sprint performance [17]. Again a similar gain in fat mass was seen in recreationally active college age males during the lockdown, with no decreases in performance seen [18]. Although there was no measure of athletic performance in the current study, previous studies have shown that changes in fat mass are inversely related to athletic performance [3,11,12,19,20]. The presence of such conflicting findings suggests that more research is needed to fully understand the impact of COVID-19 lockdowns on body composition and health-related behaviors.

Question data in the current study showed that the majority of athletes had a decrease in both quality and quantity of PA. On the other hand, a majority reporting that they consumed a greater amount of food but also that the quality of their diet was higher during the COVID-19 lockdown. Studies looking at similar outcomes in non-athletes also showed an increase in obesogenic diet behaviors and a decrease in PA due to COVID-19 [16,18,21–24]. The decrease in PA seen could be explained by the mass closure of gyms due to the pandemic and the resulting surge in the purchases of home fitness equipment. This situation led to long waits for equipment, causing many individuals to resort to bodyweight workouts or to not exercise at all. A study in rugby players found similar results with their athletes increasing both the quality and quantity of their diet during this time period [24]. In the current study none of the changes in diet or PA were associated with changes in body composition. This could be partially due to the unvalidated nature of our survey and the brief, single question measures of the relatively complex constructs of the amount and quality of diet and PA. Another possibility could be that while athletes increased their food intake they also increased the quality of their diet, which may have helped mitigate any adverse changes caused by an increase in food intake and decrease in physical activity.

There are a couple explanations that could be driving this change of diet behavior during COVID versus at school. Studies have shown that during college it can be hard for students to eat well due to access and time constraints [25–27]. These issues would be higher in college athletes due to extra demands on time, making it harder for them to eat how they would like. The decreases in time commitments and possible increase in access could be driving the changes in eating behaviors observed in our study. Another explanation could be that the home environment with parents may provide a more positive food environment for athletes compared to on-campus living (e.g., parent preparing meals, greater financial access to foods, cooking appliance, etc.). Changes in the food environment were observed in the general college age individuals and drove changes in this population [25]. For athletes being at home due to the structure, access, and time could lead to the reported changes in diet in our study, mitigating some of the negative effects seen in other populations due to the COVID-19 Lockdown.

A strength of our study is that participant sex distribution was approximately equal, and included several different sports which supports the generalizability of these results across sports and athletes. The study was limited due to its retrospective nature and the fact that athletes had been back on campus for a variable amount of time before being tested and surveyed. Regardless of this we were able to pair our body composition data with survey data which few studies have been able to do during the COVID-19 Pandemic. Due to the uniqueness of the situation, our survey had not previously been validated but it was based off of questions asked in previously validated surveys. BIA is both a strength and limitation of this study. It is strength due to its ability to detect changes in body composition as well as weight. It is a limited, however, because BIA can be influenced by hydration status and electrolyte status. However, the measurements were collected in a fasting-state in the morning on both occasions to partially mitigate this limitation.

Overall, our study found that body composition changed over the COVID-19 Lockdown in a sex-specific manner with decreases in adiposity for male athletes and increases in adiposity for female athletes. Together with the existing literature about body composition changes during breaks in training, these results will inform practitioners about how body composition is impacted by planned and unplanned interruptions to training for male and female athletes. Athletic programs should be aware of unanticipated interruptions and have plans in place to support athletes during these interruptions. Future studies should prospectively evaluate whether these body composition trends are apparent during usual breaks for college athletes, and use validated methods to objectively evaluate diet and behavior change during breaks.

## Figures and Tables

**Table 1:** Descriptive Characteristics of Athletes included in the COVID-19 analyses (n = 77). Data are mean ± SD.

	Male	Baseball	Men’s Soccer	Female	Women’s Basketball	Softball	Beach Volleyball
N	43	24	19	34	8	16	10
AGE (y/o)	20.30 ± 1.26	20.79 ± 1.14	19.10 ± 1.37	19.50 ± 1.26	19.63 ± 0.74	19.69 ± 1.40	19.10 ± 1.37
HT (in)	72.34 ± 2.80	73.48 ± 2.26	70.89 ± 2.79	68.09 ± 2.93	70.88 ± 2.30	66.47 ± 2.60	68.45 ± 2.11
ΔBMI	−0.21 ± 0.91	−0.18 ± 1.07	−0.25 ± 0.68	0.23 ± 1.2	0.24 ± 1.17	0.39 ± 1.26	−.001 ± 0.126
ΔFMI	−0.22 ± 0.50	−0.26 ± 0.57	−0.18 ± 0.42	0.28 ± 0.91	0.35 ± 0.92	0.35 ± 0.93	0.13 ± 0.94
ΔMMI	0.006 ± 0.66	0.07 ± 0.77	−0.07 ± 0.49	−0.04 ± 0.45	−0.11 ± 0.52	0.05 ± 0.46	−0.13 ± 0.41

**Table 2:** Results of unadjusted ANOVA for ΔBMI and ANCOVA for ΔFMI and ΔMMI.

	F value	P value	Male Mean ± SD	Female Mean ± SD
ΔFMI	11.21	< 0.01	−0.22 ± 0.50	0.28 ± 0.91
ΔBMI	3.43	0.07	−0.21 ± 0.91	0.23 ± 1.2
ΔMMI	1.53	0.22	0.006 ± 0.66	−0.04 ± 0.45

**Table 3:** Descriptive characteristics of Self-Reported change in Quality and Quality of diet and physical activity.

Question	Response	Total	Male	Female
Quality of diet	Got worse	20 (27%)	11 (55%)	9 (45%)
	No change	18 (25%)	12 (67%)	6 (33%)
	Got Better	35 (48%)	20 (57%)	15 (43%)
Quantity of diet	Ate more	36 (50%)	24 (67%)	12 (33%)
	No Change	20 (28%)	11 (55%)	9 (45%)
	Ate less	16 (22%)	7 (44%)	9 (56%)
Quality of PA	Got worse	39 (54%)	23 (59%)	16 (41%)
	No change	19 (26%)	11 (58%)	8 (42%)
	Got Better	14 (20%)	8 (57%)	6 (43%)
Quantity of PA	Did more	17 (23%)	10 (59%)	7 (41%)
	No Change	20 (28%)	15 (75%)	5 (25%)
	Did less	35 (49%)	18 (51%)	17 (49%)

**Table 4:** Adjusted ANCOVA for ΔBMI.

Variable	F Value	P Value
Quality of Diet	0.62	0.54
Quantity of Diet	1.91	0.16
Quality of PA	0.11	0.89
Quantity of PA	0.06	0.94
Sex	1.15	0.29

**Table 5:** Adjusted ANCOVA for ΔMMI.

Variable	F Value	P Value
ΔFMI	6.10	0.02
Quality of Diet	1.99	0.15
Quantity of Diet	1.65	0.20
Quality of PA	0.72	0.49
Quantity of PA	0.39	0.68
Sex	2.38	0.13

**Table 6:** Adjusted ANCOVA for ΔFMI.

Variable	F Value	P Value
ΔMMI	6.10	0.02
Quality of Diet	3.13	0.06
Quantity of Diet	1.58	0.22
Quality of PA	0.61	0.55
Quantity of PA	0.57	0.57
Sex	7.74	0.01

## Appendix 1

1. How do you feel the quality of your diet changed over the course of lockdown compared to your usual diet at UAB? (select one)
  - A lot worse
  - Slightly worse
  - No change
  - Slight better
  - A lot better
2. How do you feel the quantity of your diet changed over the course of lockdown compared to your usual diet at UAB? (select one)
  - A lot more
  - Slightly more
  - No change
  - Slight less
  - A lot less
3. How do you feel the quality of your physical activity changed over the course of lockdown compared to your usual physical activity at UAB? (select one)
  - A lot worse
  - Slightly worse
  - No change
  - Slight better
  - A lot better
4. How do you feel the quantity of your physical activity changed over the course of lockdown compared to your usual physical activity at UAB? (select one)
  - A lot more
  - Slightly more
  - No change
  - Slight less
  - A lot less
